# Nanocomposite Conductive Bioinks Based on Low-Concentration GelMA and MXene Nanosheets/Gold Nanoparticles Providing Enhanced Printability of Functional Skeletal Muscle Tissues

**DOI:** 10.1021/acsbiomaterials.1c01193

**Published:** 2021-11-22

**Authors:** Selwa Boularaoui, Aya Shanti, Michele Lanotte, Shaohong Luo, Sarah Bawazir, Sungmun Lee, Nicolas Christoforou, Kamran A. Khan, Cesare Stefanini

**Affiliations:** † Department of Biomedical Engineering, Khalifa University of Science and Technology, 127788, Abu Dhabi, UAE; ‡ Advanced Digital and Additive Manufacturing (ADAM) Center, Khalifa University of Science and Technology, 127788, Abu Dhabi, UAE; § Healthcare Engineering Innovation Center (HEIC), Khalifa University of Science and Technology, 127788, Abu Dhabi, UAE; ∥ Department of Civil Infrastructure and Environmental Engineering, Khalifa University of Science and Technology, 127788, Abu Dhabi, UAE; ⊥ Rare Disease Research Unit, Pfizer Inc., 610 Main Street, Cambridge, Massachusetts 02139, United States; # Department of Aerospace Engineering, Khalifa University of Science and Technology, 127788, Abu Dhabi, UAE; ∇ The Biorobotics Institute, Scuola Superiore Sant’Anna, Piazza Martiri della Libertà, 33, 56127 Pisa, PI, Italy

**Keywords:** 3D bioprinting, GelMA, MXene nanosheets, gold nanoparticles, skeletal tissue

## Abstract

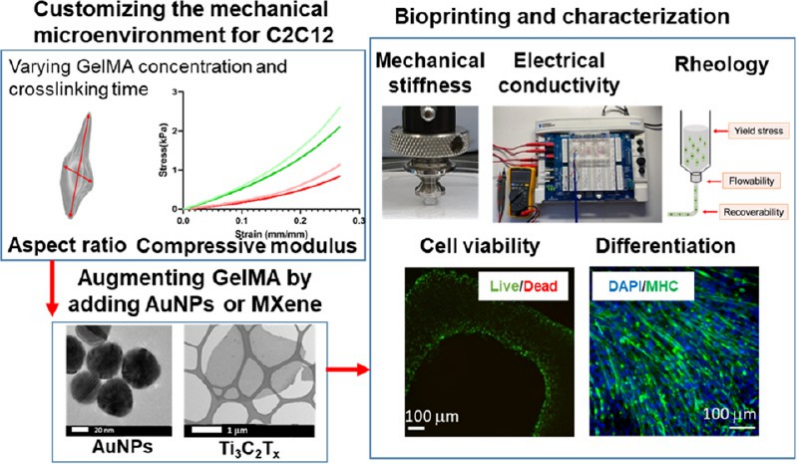

There is a growing need to develop novel well-characterized biological inks (bioinks) that are customizable for three-dimensional (3D) bioprinting of specific tissue types. Gelatin methacryloyl (GelMA) is one such candidate bioink due to its biocompatibility and tunable mechanical properties. Currently, only low-concentration GelMA hydrogels (≤5% w/v) are suitable as cell-laden bioinks, allowing high cell viability, elongation, and migration. Yet, they offer poor printability. Herein, we optimize GelMA bioinks in terms of concentration and cross-linking time for improved skeletal muscle C2C12 cell spreading in 3D, and we augment these by adding gold nanoparticles (AuNPs) or a two-dimensional (2D) transition metal carbide (MXene nanosheets) for enhanced printability and biological properties. AuNP and MXene addition endowed GelMA with increased conductivity (up to 0.8 ± 0.07 and 0.9 ± 0.12 S/m, respectively, compared to 0.3 ± 0.06 S/m for pure GelMA). Furthermore, it resulted in an improvement of rheological properties and printability, specifically at 10 °C. Improvements in electrical and rheological properties led to enhanced differentiation of encapsulated myoblasts and allowed for printing highly viable (97%) stable constructs. Taken together, these results constitute a significant step toward fabrication of 3D conductive tissue constructs with physiological relevance.

## 1 Introduction

Tissue engineering is a multidisciplinary field that utilizes principles of cellular biology, mechanobiology, engineering, materials science, and medicine to develop engineered tissues that can ultimately restore, maintain, or improve damaged body tissues. To achieve this, different tissue fabrication techniques have been proposed, amongst which three-dimensional (3D) bioprinting has stood up as a promising technology.^1−3^ 3D bioprinting offers the ability to customize tissue’s material, shape, and organization, allowing the mimicking of the hierarchical structure of native tissues.^4,5^ The process of 3D bioprinting involves preparation of a bioink with biomimetic extracellular matrix (ECM) components, signaling molecules, and cellular elements followed by actual printing of the bioink, layer by layer, to achieve desired tissue construct, and finally, maintenance of the tissue construct in growth media. The development of 3D bioprinted tissue constructs with physiological relevance has been proven to be challenging, requiring sophisticated optimization of in vitro tissue characteristics, including mechanical, biochemical, and electrical factors. Therefore, a significant effort has been devoted to developing and characterizing novel bioinks with enhanced properties. A bioink optimal for extrusion bioprinting should (1) be biocompatible, maintaining cell viability and allowing proper cell adhesion, proliferation, and differentiation, (2) be biomimetic, possessing ECM components comparable to those found in vivo, (3) have appropriate viscosity allowing extrusion and shape recovery of the printed filaments, and (4) exhibit appropriate shear-thinning properties to maintain cell viability and shape fidelity.^3,6−8^

In the literature, several attempts have been made to enhance the properties of inks for bioprinting applications focusing on either one or combination of the following properties: printability, biocompatibility, electrical properties, or mechanical properties.^9−11^ Here, we adopted a unique approach in which we first identified an ink compatible with skeletal muscle cells, supporting cell spreading and preserving cell viability, then sought to optimize its electrical and rheological properties. Among the different available bioinks, GelMA-based bioinks hold great potential, which is attributed to their superior biocompatibility and broadly tunable mechanical properties.^12^ GelMA is synthesized by the reaction of gelatin with methacrylic anhydride (MA) and is covalently cross-linked by UV light in the presence of a photoinitiator to form stable constructs. GelMA hydrogels have properties that closely resemble those in vivo due to the presence of cell-attaching sites.^13^ Although high concentrations of GelMA have been widely used as tissue-engineered scaffolding materials that are printed and then seeded with cells, only low-concentration GelMA hydrogels (≤5% w/v) are suitable as cell-laden bioinks to enable high cell viability, elongation, and migration. However, low concentrations of GelMA still lead to very poor printability and limited layer stacking ability, thus limiting their use.^13^

Several studies have shown that bioink printability is governed by bioink composition and printing parameters.^14,15^ To enhance the printability and shear-thinning properties of hydrogels in general, incorporation of different additives such as nanoparticles and 2D materials has been employed and has, so far, demonstrated excellent results.^16−18^ For GelMA hydrogels, in particular, efforts have been devoted to high concentrations rather than low ones, although low concentrations produce better cell-laden constructs.^10^

MXenes are a family of 2D transition metal carbides or nitrides with attractive features including conductivity, mechanical flexibility, and hydrophilicity.^19^ Ti_3_C_2_T_*x*_ is an extensively studied MXene with high hydrophilicity, electrical conductivity, and stability.^20^ Ti_3_C_2_T_*x*_ has been used in water desalination, photocatalysis, and biosensing.^21−23^ Despite its utilization in different biomedical applications, the effect of incorporating MXene in bioprinted bioinks has been poorly explored.^24^ In the literature, there is only one study conducted by Rastin et al. in which MXene was incorporated in a hyaluronic acid/alginate hydrogel. The results of the study showed promising potential of MXene in 3D bioprinting.^25^

Similarly, AuNPs are attractive nanoparticles with wide applications in biology and medicine. They have been explored in drug delivery, tumor imaging, and cancer therapy.^26,27^ AuNPs can be tuned in terms of shape and size, functionalized, and integrated in different scaffolding materials to be used for tissue engineering and regenerative medicine applications.^28^ Specifically, in bioprinting, Zhu et al. developed a bioink composed of gold nanorods, GelMA, and alginate, and reported enhanced functionality of printed cardiac tissue constructs.^29^ However, efforts are still limited in terms of exploring the effect of gold nanoparticles on bioink printability and 3D bioprinted constructs properties.

In this study, the GelMA concentration and cross-linking time were optimized to support spreading of differentiating skeletal muscle cells in 3D. Two GelMA-based bioinks composed of low-concentration GelMA with spherical gold nanoparticles or with MXene nanosheets were developed, and their biological, mechanical, conductive, and rheological properties were evaluated to investigate their suitability for skeletal muscle extrusion-based bioprinting.

## 2 Materials and Methods

### 2.1 Cell Culture

The murine myoblast cell line, C2C12, was obtained from Addexbio Technologies. Cells were maintained in Dulbecco’s modified Eagle medium (DMEM) containing 4.5 g/L d-glucose (Sigma-Aldrich) and supplemented with 10% fetal bovine serum (FBS; Sigma-Aldrich), 25 μg/mL gentamicin (Sigma-Aldrich), 2 mM l-glutamine (Sigma-Aldrich), nonessential amino acids (Hyclone), and 1 mM sodium pyruvate (Hyclone). Cells were cultured at 37 °C in a humidified atmosphere with 5% CO_2_. Cells were routinely passaged to avoid maximal confluency and subsequent unwarranted differentiation. For all experiments, cells were detached from culture flasks using trypsin/EDTA (Hyclone). To induce differentiation, growth media were replaced by low-serum media (supplemented with 2% FBS instead of 10% FBS).

### 2.2 GelMA Preparation

A GelMA lyophilizate and a LAP photoinitiator (PI) were both purchased from CELLINK, Sweden. A 0.1% PI solution was prepared by adding the required amount of PI to deionized (DI) water, and then the solution was heated to 50 °C until fully dissolved. Then, the solution was added to the preweighed GelMA lyophilizate to prepare GelMA of the following concentrations: 2, 4, and 6% (w/v). The mixture was stirred at 50 °C for 1 h.

### 2.3 Cell Morphology

To examine the morphology/spreading of C2C12 cells in GelMA hydrogels, cells were encapsulated in 2% GelMA, 4% GelMA, and 6% GelMA, and then exposed to UV light (wavelength 365 nm) for either 2 or 4 min, allowed to grow for 7 days, and imaged using a microscope on days 1 and 7. Quantitative analysis of cellular elongation 1 day post encapsulation was performed using ImageJ.^30,31^ The aspect ratio of cells, which is defined as the length of the major access across the nucleus of the cell divided by the length of the minor axis, was measured, given that the cell is approximated to be an ellipse.

Furthermore, on day 7, cells were immunostained with phalloidin following the same fixation, permeabilization, and staining steps described in Section 2.12.

### 2.4 MXene Synthesis

Ti_3_C_2_T*_x_* (MXene) was synthesized following the optimized MILD method, in which Al layers were selectively etched from Ti_3_AlC_2_.^32^ Briefly, the etchant was formed by adding 3.2 g of LiF powder (Sigma-Aldrich) to 40 mL of a 9 M HCl solution (Sigma-Aldrich). Next, 2 g of Ti_3_AlC_2_ powder (Carbon-Ukraine) was immersed in the etchant and stirred for 24 h at room temperature. Then, the etchant was washed with deionized (DI) water for several cycles (10 min for each cycle) via centrifugation at 5500 rpm until pH > 6. This was followed by collection of a stable dark green Ti_3_C_2_T*_x_* supernatant by prolonged sonication and 1 h centrifugation at 3500 rpm. The obtained Ti_3_C_2_T*_x_* solution contained single-layer or few-layer Ti_3_C_2_T*_x_*.

### 2.5 MXene and AuNP Characterization

Synthesized MXene nanosheets were characterized using X-ray diffraction (XRD) and transmission electron microscopy (TEM). XRD patterns were obtained using a PANalytical Empyrean XRD system, which employs copper K-α radiation and a scan step of 0.1° with 0.5 s per step. TEM was performed on a Titan TEM system using an acceleration voltage of 300 kV. TEM samples of Ti_3_C_2_T*_x_* were prepared by placing two drops of a diluted Ti_3_C_2_T*_x_* solution on a lacey carbon-coated copper grid (Agar Scientific Ltd.). Selected area electron diffraction (SAED) patterns were also acquired to determine the crystal structure of the samples. AuNPs with a 50 nm diameter purchased from Sigma-Aldrich were imaged using TEM.

### 2.6 Biocomposite Ink Preparation

To prepare 2% GelMA containing AuNPs or MXene nanosheets, a concentration of GelMA higher than 2% was initially prepared, and then diluted with deionized (DI) water containing either AuNPs or MXene to obtain following hydrogel mixtures: 2% GelMA containing five different concentrations of MXene: 0.05, 0.1, 0.5, 1, and 3 mg/mL MXene or 2% GelMA containing two different concentrations of AuNPs: 0.05 and 0.1 mg/mL AuNPs. All prepared biocomposite hydrogels were cross-linked via exposure to UV light (wavelength 365 nm) for 4 min.

### 2.7 Mechanical Characterization

#### 2.7.1 Mechanical Stiffness

Compressive stiffness of different GelMA bioinks (Table 1) under unconfined compression was determined using an Instron 5948 MicroTester. In brief, cylindrical specimens of formulated GelMA, 4 mm in diameter and 6 mm in height, were prepared using a custom-built PTFE (Teflon) mold. Next, the specimens were mounted on a testing machine and subjected to compressive stress. In particular, specimens were tested at room temperature with 0.01 N preload force and a 0.75 mm/min strain rate. Finally, the compressive modulus of different specimens was calculated from the initial linear region of the obtained stress–strain curve.

**Table 1:** Tested GelMA-Based Bioinks for the Mechanical Test

concentration	UV time exposure	
2% GelMA		4 min
4% GelMA	2 min	4 min
6% GelMA	2 min	4 min
2% GelMA-0.05 mg/mL AuNPs		4 min
2% GelMA-0.05 mg/mL MXene		4 min

#### 2.7.2 Swelling Ratio

To study the swelling behavior of the different GelMA bioinks (pure GelMA, GelMA with AuNPs, GelMA with MXene), the hydrogels were hydrated and then their mass swelling ratio was determined. In brief, GelMA hydrogels were first weighed to determine the dehydrated mass (D_m_). Next, GelMA hydrogels were hydrated with PBS and incubated for 24 h. After 24 h, hydrogels were removed from PBS and weighed to determine the hydrated mass (H_m_). Finally, the mass swelling ratio was calculated by the equation
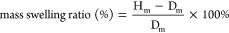
1

### 2.8 Electrical Characterization

To assess the electrical conductivity of the different GelMA bioinks (pure GelMA, GelMA with AuNPs, GelMA with MXene), a four-terminal sensing method was adopted. In brief, thin films of GelMA hydrogels were coated on glass slides and then attached to a custom-built four-terminal sensing device. Next, a constant current was passed through the outer terminals of the device and the voltage was measured through the inner terminals. Consequently, resistivity (ρ) was calculated as
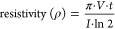
2where *I* is the applied current, *V* is the measured voltage, and *t* is the sample thickness (300 μm).^33^ Finally, the electrical conductivity (σ) was calculated as the inverse of resistivity.

### 2.9 Rheological Evaluation

The evaluation of the rheological behavior and properties of different GelMA-based hydrogels was performed using an MCR 302 rheometer (Anton Paar, Germany) equipped with a 25 mm parallel plate measuring system. The measuring system was coupled with a Peltier cell for temperature control. Hydrogels were placed on the bottom plate, gently squeezed by the top plate to reach a resting position of 0.5 mm gap, and excess material was trimmed out with a metal spatula. Once in place, hydrogels’ thermal equilibrium was achieved by keeping the sample at the testing temperature for 5 min. Shear rate sweep, shear stress sweep, and time sweep in the oscillatory mode were performed to obtain information on the materials’ behavior during different stages of the application process.

Shear rate sweep tests were performed in the range between 10^–3^ and 10^3^ s^–1^. Pure GelMA was tested at 4, 10, and 20 °C, while other hydrogels were tested at 10 °C for comparative purposes. During the test, viscosity values were recorded as a function of the applied shear rate to evaluate the shear-thinning and flow behaviors of the hydrogels. Shear stress sweep tests were performed at 10 °C and 1 Hz using increasing shear stress levels in the range 1–100 Pa. The loss modulus (*G*′) and the storage modulus (*G*″) of the hydrogels were recorded with the goal of identifying the *G*′–*G*″ crossover point (yield stress) to be used as an indicator of the change in the viscoelastic behavior of the hydrogels. Finally, the time sweep tests in the oscillatory mode were performed by alternating 60 s intervals at low (1%) and high (100%) strain levels. Storage moduli were recorded during each test. For comparison purposes on the recoverability of the hydrogels, data were normalized using the first initial storage modulus obtained.

### 2.10 Bioprinting

Bioinks containing C2C12 (5 × 10^6^ cells per mL) were prepared and transferred to a 3 mL UV shielding cartridge, capped with a 27G conical nozzle and placed in the printing head (precooled to 10 °C) of Inkredible+ bioprinter (Cellink). Consequently, dots of different bioink constitutions were printed in a 96-well plate to assess the effect of bioprinting on cell viability. With the dot shape, the cell viability is only affected by the printing process and not by the other construct properties, such as construct porosity.

Furthermore, tubular and mesh structures were imported into Slic3r, sliced, and converted into G-codes using Cellink HeartWare software. G-codes were loaded into the Inkredible+ bioprinter. Printing was performed at a speed of 5 mm/s. For cross-linking, samples were subjected to UV light for 4 min.

### 2.11 Cell Viability in Bulk and Bioprinted Structures

To assess the cellular toxicity of the materials added to GelMA hydrogels, i.e., AuNPs and MXene, viability of cells encapsulated in 2% GelMA hydrogels containing either 0.05 or 0.1 mg/mL AuNPs or MXene was determined via Live/Dead assay. Cell viability was assessed on day 1 and day 7 in which Calcein AM (green dye) was used to stain live cells and ethidium homodimer-1 (red dye) was used to stain dead cells. Cell viability was calculated as
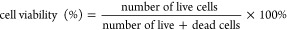
3

Cells in pure 2% GelMA and in 75% ethanol-treated pure 2% GelMA were considered as the positive and negative controls, respectively. The number of live and dead cells was determined using ImageJ.

To assess the effect of bioprinting on cell survival, cells were encapsulated in the different GelMA bioinks, printed using A 27G nozzle (200 μm in diameter) and assessed for viability via Live/Dead assay, as previously described. Cells encapsulated in GelMA hydrogels but not printed were regarded as the control.

### 2.12 Myotube Formation Analysis with Immunocytochemistry

To investigate the potential of myogenic differentiation of C2C12 cells encapsulated in different GelMA hydrogels, cells encapsulated in pure GelMA, GelMA with AuNPs, and GelMA with MXene were incubated in differentiation media (DMEM containing 2% FBS) for 7 days, and then immunostained for the myosin heavy chain (MHC), a protein expressed during myotube formation. In specific, cells were fixed with 4% paraformaldehyde, permeabilized with 0.1% Triton X-100, blocked with a 5% BSA solution, incubated with a primary antibody at 4 °C overnight, incubated with a secondary antibody at room temperature for 1 h, imaged using a fluorescence microscope, and finally analyzed with ImageJ. Analysis performed using ImageJ included determining the fusion index (number of nuclei in MHC-positive cells with more than two nuclei divided by the total number of nuclei), the length of myotubes, and the diameter of myotubes. The number of myotubes analyzed was ∼30.

### 2.13 Statistical Analysis

Statistical analysis was performed using Microsoft Excel and GraphPad Prism 9. Student’s *t*-test was used to determine the significance with *p* < 0.05 taken as significant. Experiments were performed in triplicates.

## 3 Results and Discussion

### 3.1 Effect of Bioink Stiffness on Cell Morphology

Aiming to find the most favorable mechanical microenvironment for C2C12 spreading and elongation, GelMA bioinks with different stiffnesses were prepared. C2C12 cells differentiate to form skeletal muscle myotubes, and they are commonly used to study skeletal muscle differentiation and regeneration. Cell spreading and elongation are key for cell to cell communication, which, in turn, is necessary for cell viability, proliferation, and fusion.^34^ The cellular aspect ratio was used as a cell morphology descriptor that quantitatively evaluates the cell ability to spread and elongate. Bioink stiffness was altered by two variables: the GelMA concentration and GelMA cross-linking time. C2C12 myoblasts were encapsulated in GelMA bioinks prepared at different concentrations and cross-linked for 2 min or 4 min via UV exposure, and their morphology was examined.

**Figure 1:**
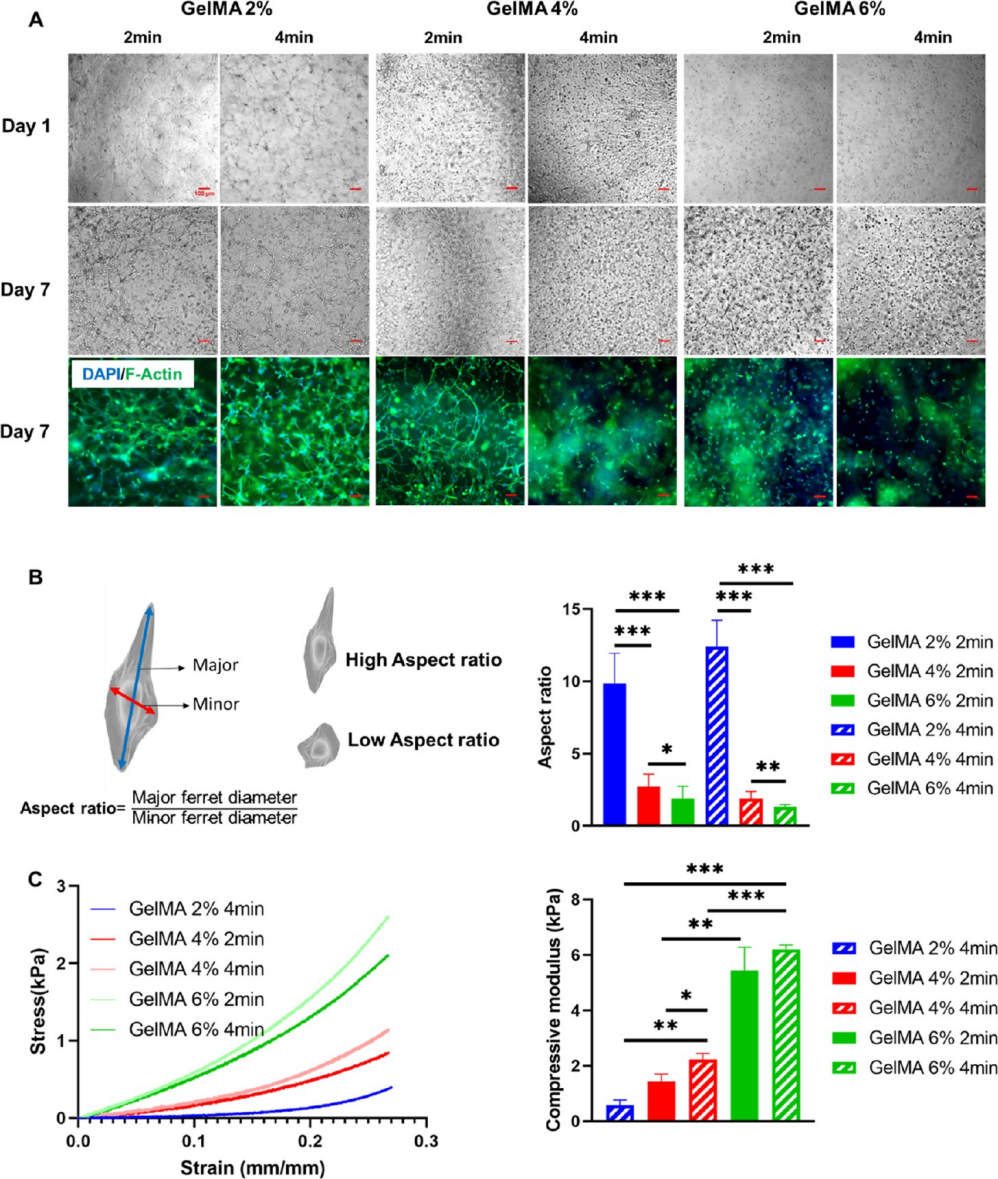
Effect of hydrogel stiffness on the cell morphology. (A) Microscopy images (scale bar = 100 μm) and (B) aspect ratio of cells encapsulated in 2, 4, and 6 GelMA hydrogels cross-linked for either 2 or 4 min using UV light. (C) Stress–strain curves and associated compressive moduli for 2, 4, and 6% GelMA hydrogels cross-linked for either 2 or 4 min using UV light, except for 2% GelMA cured for 2 min as it was difficult to obtain a stable cylinder for the test to be performed.

Bright-field images of encapsulated C2C12 in the cross-linked hydrogel on days 1 and 7 along with stained actin filaments on day 7 are shown in Figure 1A. After 1 day of encapsulation, cells in 2% GelMA demonstrated better spreading and elongation compared to cells encapsulated in 4 and 6% GelMA, which were mostly round in shape. However, by day 7, cells encapsulated in 4% GelMA and cured for 2 min showed an enhanced radial branching morphology compared to day 1, but cells encapsulated in higher UV exposure or a higher GelMA concentration remained almost circular and localized in clusters. Cells in 2% GelMA showed the highest elongation with an aspect ratio of 9.88 ± 2.08 and 12.40 ± 1.82 for 2 and 4 min cross-linking time, respectively, as shown in Figure 1B. Limited cell elongation was observed in 4% GelMA cured for 2 min with an aspect ratio of 2.72 ± 0.86. Increasing both the concentration and cross-linking time of the hydrogels decreased the aspect ratio reaching 1.31 ± 0.16 for 6% GelMA cross-linked for 4 min.

Myoblasts are known to sense the stiffness of the environment and respond accordingly; it has been previously proven that substrate stiffness significantly affects skeletal muscle cells’ adhesion, spreading, proliferation, and differentiation in 2D.^35,36^ However, the effect of stiffness can be remarkably different when cells are cultured in 3D.^37^ An unconfined compression test was performed to characterize the mechanical properties of cross-linked GelMA hydrogels. Figure 1C shows the stress–strain curves for the tested hydrogels along with the calculated compressive moduli. Increasing either the GelMA concentration or UV exposure time led to a significant increase in the compressive modulus, starting with 0.58 ± 0.18 kPa for 2% GelMA cross-linked for 4 min to 6.21 ± 0.14 kPa for 6% GelMA cross-linked for 4 min. This increase in the compressive modulus is an indicator of the increased amount of cross-linking in the gel, forming a tighter network, which, in turn, restricted cell access to adhesion sites.

Overall, we can conclude that GelMA bioinks with low stiffness support C2C12 spreading in 3D, and since, 2% GelMA cross-linked for 4 min was found to induce the best elongation and spreading of embedded cells, it was selected for further studies and denoted from here after “pure GelMA”. GelMA hydrogels with concentrations lower than 2% could result in a better cellular aspect ratio; however, they are very hard to handle (very soft) and require prolonged exposure to UV for cross-linking, which significantly affect cell viability and functionality.

Enhancing printability, mechanical, and electrical properties of 2% GelMA is a challenge that was addressed in this study by incorporating AuNPs and MXene nanosheets into 2% GelMA and printing at a relatively low temperature.

### 3.2 Characterization of MXene Nanosheets and Gold Nanoparticles

**Figure 2:**
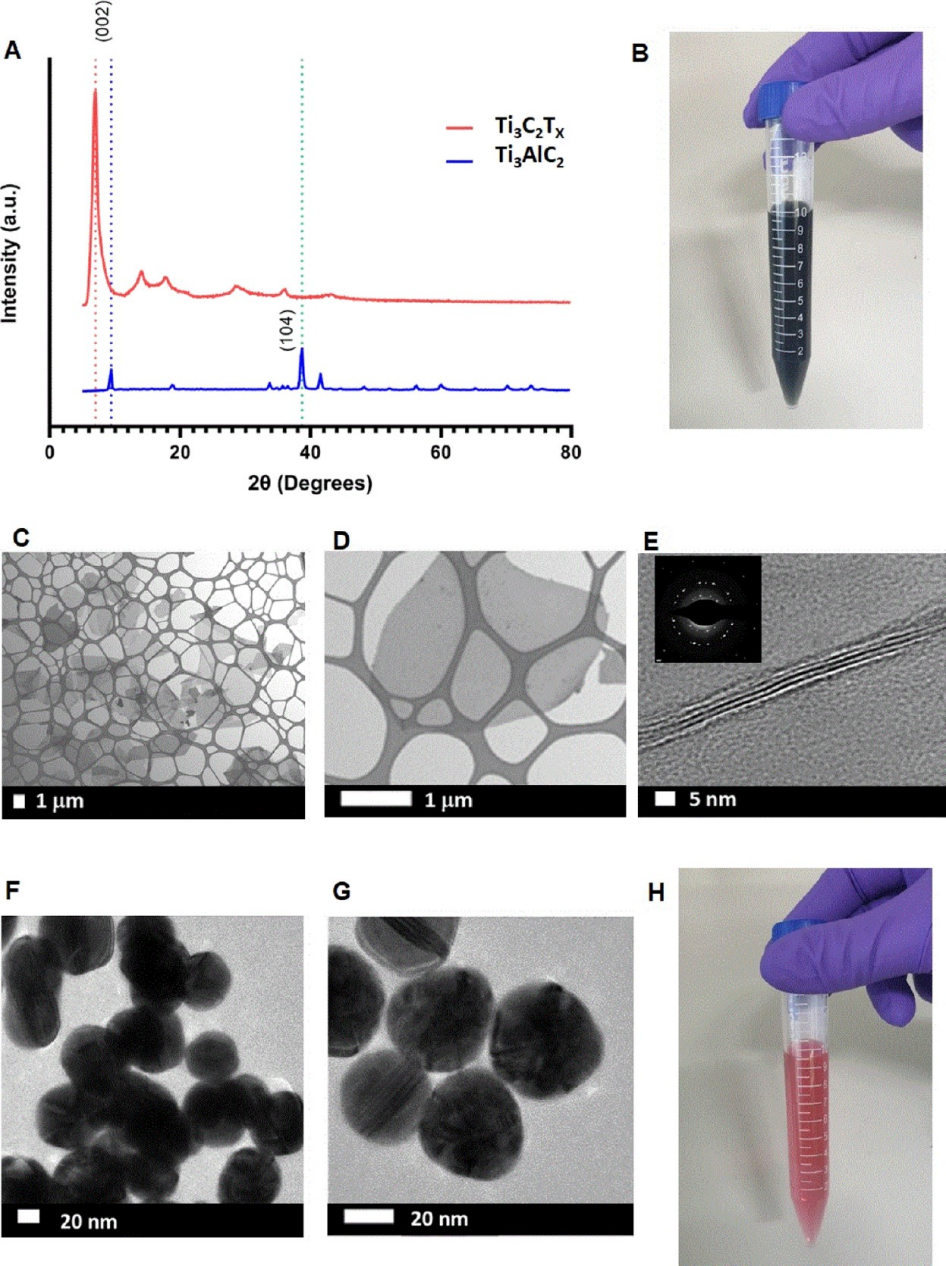
Characterization of MXene and gold nanoparticles (AuNPs). (A) XRD patterns of Ti_3_AlC_2_ and Ti_3_C_2_T*_x_*, (B) homogeneous solution of MXene in deionized water, (C) low- and (D, E) high-magnification TEM images showing the lateral size and thickness of the synthesized nanosheets (inset: SAED pattern), (F) low- and (G) high-magnification TEM images showing the diameter of the AuNPs, and (H) homogeneous solution of AuNPs in deionized water.

Ti_3_C_2_T*_x_* nanosheets were stably dispersed in DI water forming an aqueous suspension with no sediment or aggregation due to the presence of oxygen-rich functional groups such as −OH and −O, as shown in Figure 2B. XRD analysis from which we can assure the successful exfoliation and delamination of Ti_3_AlC_2_ MAX phase powder into Ti_3_C_2_T*_x_* nanosheets is exhibited in Figure 2A. Aluminum (Al) was successfully removed, as indicated by the shift of the (002) peak from 9.39° (blue dotted line) to 7.00° (red dotted line) and the disappearance of the peak of 2θ = 39° (green dotted line). In addition, a Li^+^ ion and water interaction led to an expanded Ti_3_C_2_T*_x_* layer spacing.^32^ The morphology of obtained Ti_3_C_2_T*_x_* nanosheets was investigated using TEM, as shown in Figure 2C–E. MXene nanosheets are clearly ultrathin as indicated by their transparency when deposited on the lacey carbon grid. Their lateral size is 2–3 μm and their thickness is 3–4 nm (3–4 layers). In addition, nanosheets’ crystallinity was confirmed by the SAED pattern.

Similarly, AuNPs were homogeneously dispersed in DI water, as shown in Figure 2H. High- and low-magnification TEM images of the purchased gold nanoparticle were taken, and both show that the size of the nanoparticles is around 50 nm, as shown in Figure 2F,G; based on the literature, this size does not affect cell metabolic activity if used in moderate concentrations.^38^

### 3.3 Mechanical Properties, Conductivity, and Biocompatibility of Biocomposite Inks

In the literature, GelMA viscosity and mechanical properties were improved via the incorporation of cellulose nanofibers, alginate, and gelatin and its conductivity was enhanced by mixing it with inherently conductive polymers.^39−41^ However, this study is the first to develop biocomposite inks consisting of pure low-concentration GelMA and either gold nanoparticle or MXene nanosheets and investigate their different properties.

**Figure 3:**
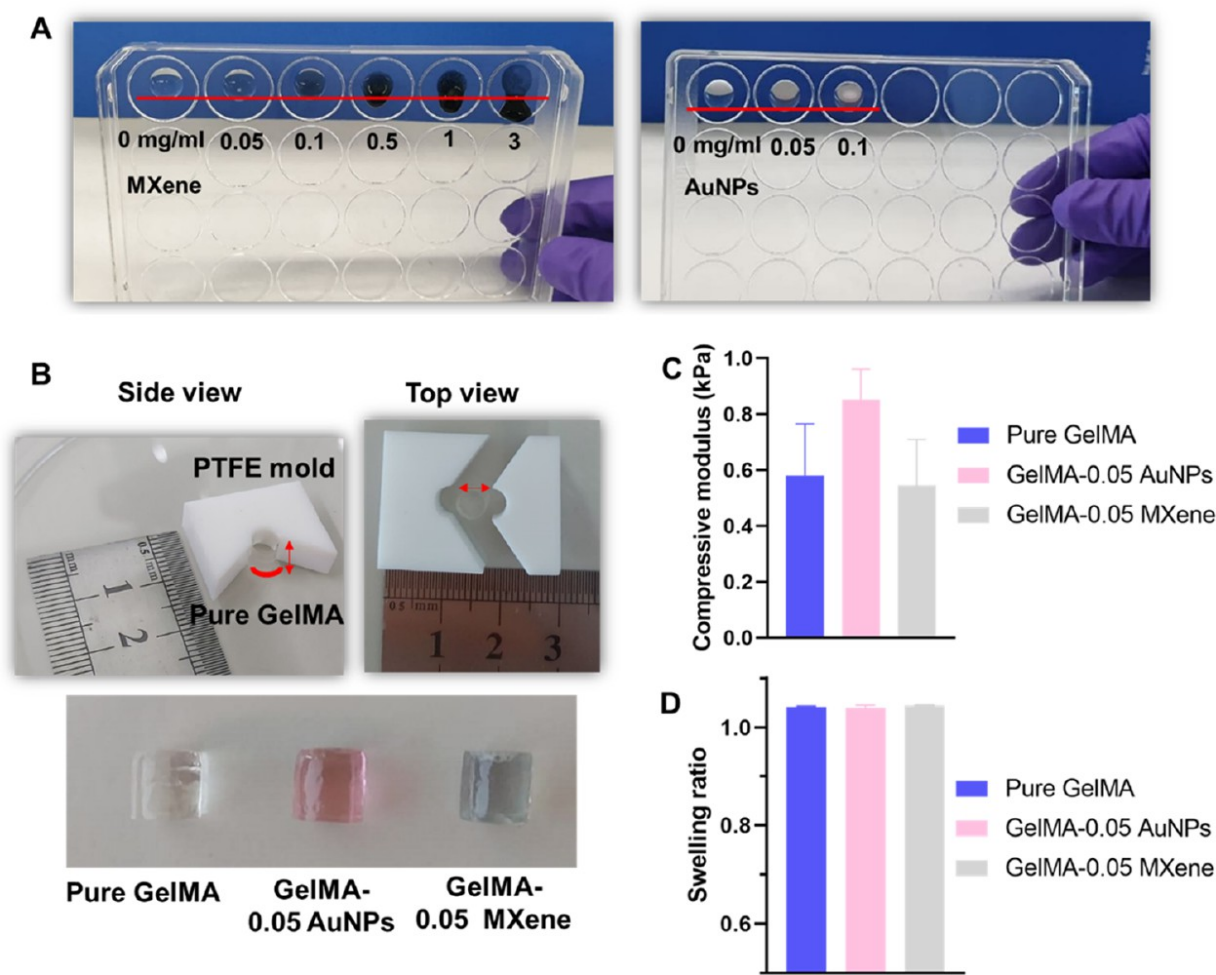
Mechanical properties of GelMA-AuNPs and GelMA-MXene hydrogels. (A) Cross-linking test for different concentrations of MXene and AuNPs incorporated in 2% GelMA hydrogels, (B) custom-built mold to obtain hydrogels in cylindrical shapes for compressive testing, (C) compressive moduli, and (D) swelling ratios of pure GelMA, GelMA-AuNPs, and GelMA-MXene hydrogels.

Biocomposite inks consisting of 2% GelMA with increasing concentrations of MXene (0.05–3 mg/mL) or with two concentrations of AuNPs (0.05 and 0.1 mg/mL) were prepared, as explained in Section 2.6. Drops of these bioinks were placed on a plate and allowed to cross-link under UV light for 4 min. Afterward, the plate was held at a 90° angle to observationally assess cross-linking, as shown in Figure 3A. Bioink drops containing up to 0.1 mg/mL either MXene or AuNPs were able to cross-link and remain stable on their loading site; however, MXene concentrations above 0.1 mg/mL hindered GelMA cross-linking, prevented formation of stable constructs, and showed bioink leakage. One reason for weak cross-linking observed with higher concentrations of MXene could be the interference of MXene functional groups (such as oxygen and hydroxyl) with GelMA functional groups (amines and hydroxyl), which, in turn, impairs the free-radical photopolymerization GelMA undergoes under UV exposure. For AuNPs, concentrations higher than 0.1 mg/mL were not tested as they result in increased UV light reflection, leading to softer gelation, as reported by Zhu et al.^29^

Cylinders of cross-linked bioinks with a 0.05 mg/mL concentration of either AuNPs or MXene were mechanically tested to further investigate the effect of AuNPs and MXene addition on bioink stiffness, as shown in Figure 3B. No significant difference in the compressive modulus was found between the tested bioink formulations, as shown in Figure 3C. This indicates that these developed bioinks will support cell elongation and spreading similar to pure GelMA. In addition, the swelling ratio of cross-linked GelMA did not change with changing bioink formulations, as shown in Figure 3D. The resultant low swelling ratio is an indicator of bioink suitability for 3D bioprinting and of enhanced fidelity of bioprinted constructs.^42^

**Figure 4:**
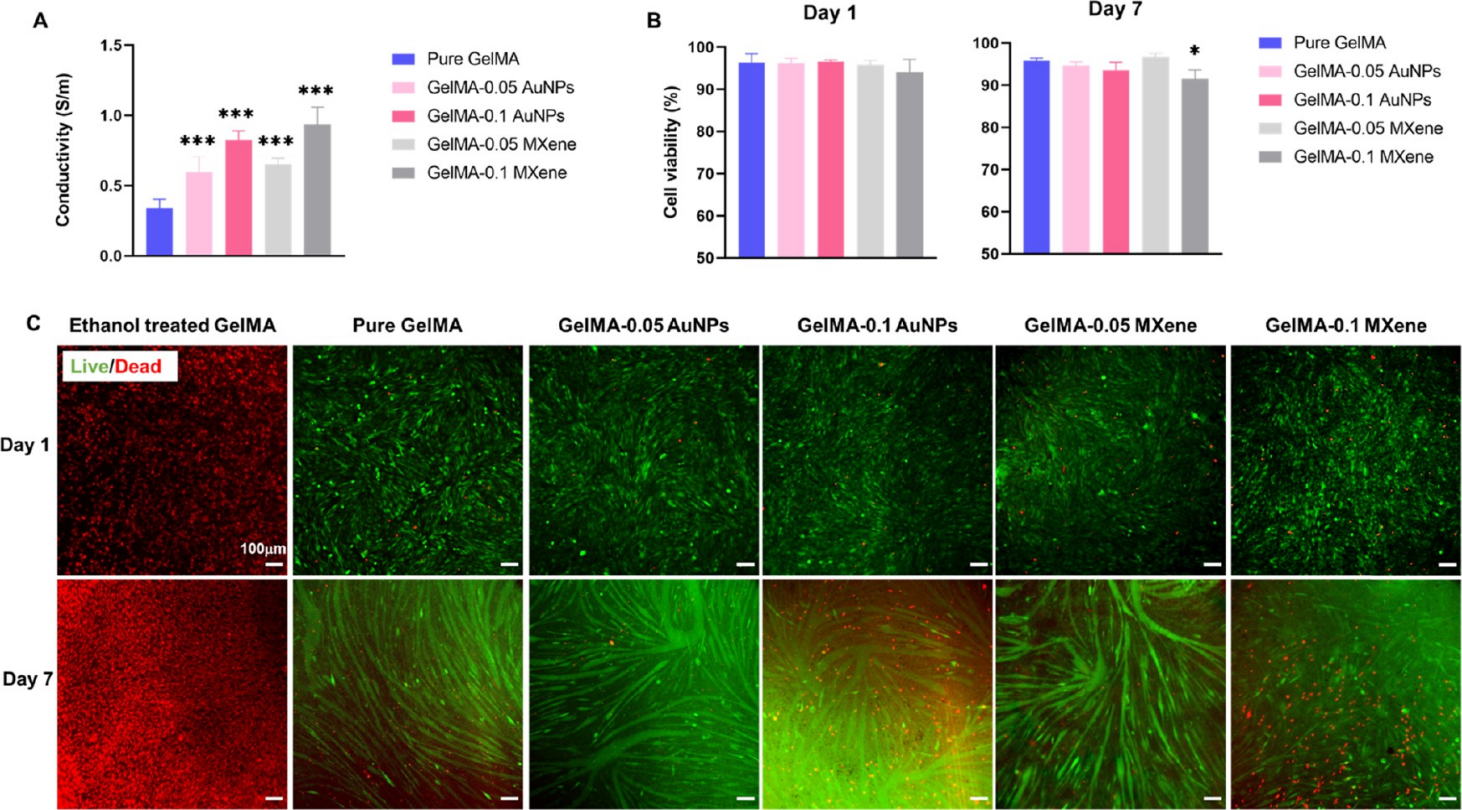
Conductivity and biocompatibility of GelMA-AuNPs and GelMA-MXene hydrogels. (A) Conductivity of pure GelMA, GelMA-AuNPs, and GelMA-MXene hydrogels; (B) cell viability; and (C) fluorescent microscopy images of C2C12 cells encapsulated in pure GelMA, GelMA-AuNPs, and GelMA-MXene hydrogels. Red cells are dead cells, while green cells are live cells.

Next, the electrical conductivity of bioinks was evaluated. Electrical conductivity is an essential property for excitable cell types such as nerve and muscle cells; electroconductive hydrogels mimic the native ECM environment, which provides electrical cues to living cells necessary for their development.^43^ In addition, such hydrogels improve electrical signal propagation upon electrical stimulation. As expected, the addition of both MXene and AuNPs endowed GelMA with conductivity, which showed an increase with increasing concentration, as shown in Figure 4A. Recent studies have demonstrated the ability of gold nanoparticles to enhance conductivity when incorporated in alginate, decellularized matrices, synthesized thiol-HEMA/HEMA, and chitosan.^33,44−46^ In accordance, our results showed that gold nanoparticles can enhance the conductivity of low-concentration GelMA to up to 0.60 ± 0.11 and 0.82 ± 0.07 S/m when 0.05 and 0.1 mg/mL AuNP concentrations were used, respectively.

MXene, with its highly conductive properties, was previously implemented in the development of conductive hydrogels such as an MXene-catalyzed poly(acrylic acid) (PAA) hydrogel, a MXene-hyaluronic acid/alginate hydrogel, and MXene-composited poly(vinyl alcohol)/poly(vinyl pyrrolidone) double-network hydrogels.^25,47,48^ In our study, MXene enhanced the conductivity of low-concentration GelMA to 0.65 ± 0.04 and 0.94 ± 0.12 S/m when 0.05 and 0.1 mg/mL concentrations were used, respectively. Conductivity values achieved by either the addition of AuNPs or MXene were in the range of electrical conductivity of the excitable tissues (0.4–0.9 S/m), which indicates that such hydrogels are biomimetic and of physiological relevance.

Subsequently, the in vitro cytotoxicity of AuNPs and MXene to C2C12 encapsulated within GelMA was assessed on day 1 and day 7 post encapsulation using a viability assay, as shown in Figure 4B,C. MXene at a concentration of 0.1 mg/mL showed a significant decrease in cell viability on day 7; however, all other formulations did not result in a decrease in cell viability over the entire week of analysis. Since the optimal results in terms of cross-linking, mechanical strength, conductivity, and cell viability (in combination) were achieved at a concentration of 0.05 mg/mL AuNPs and 0.05 mg/mL MXene, subsequent experiments were pursued using these concentrations.

### 3.4 Rheological Characterization and Printability of Biocomposite Inks

**Figure 5:**
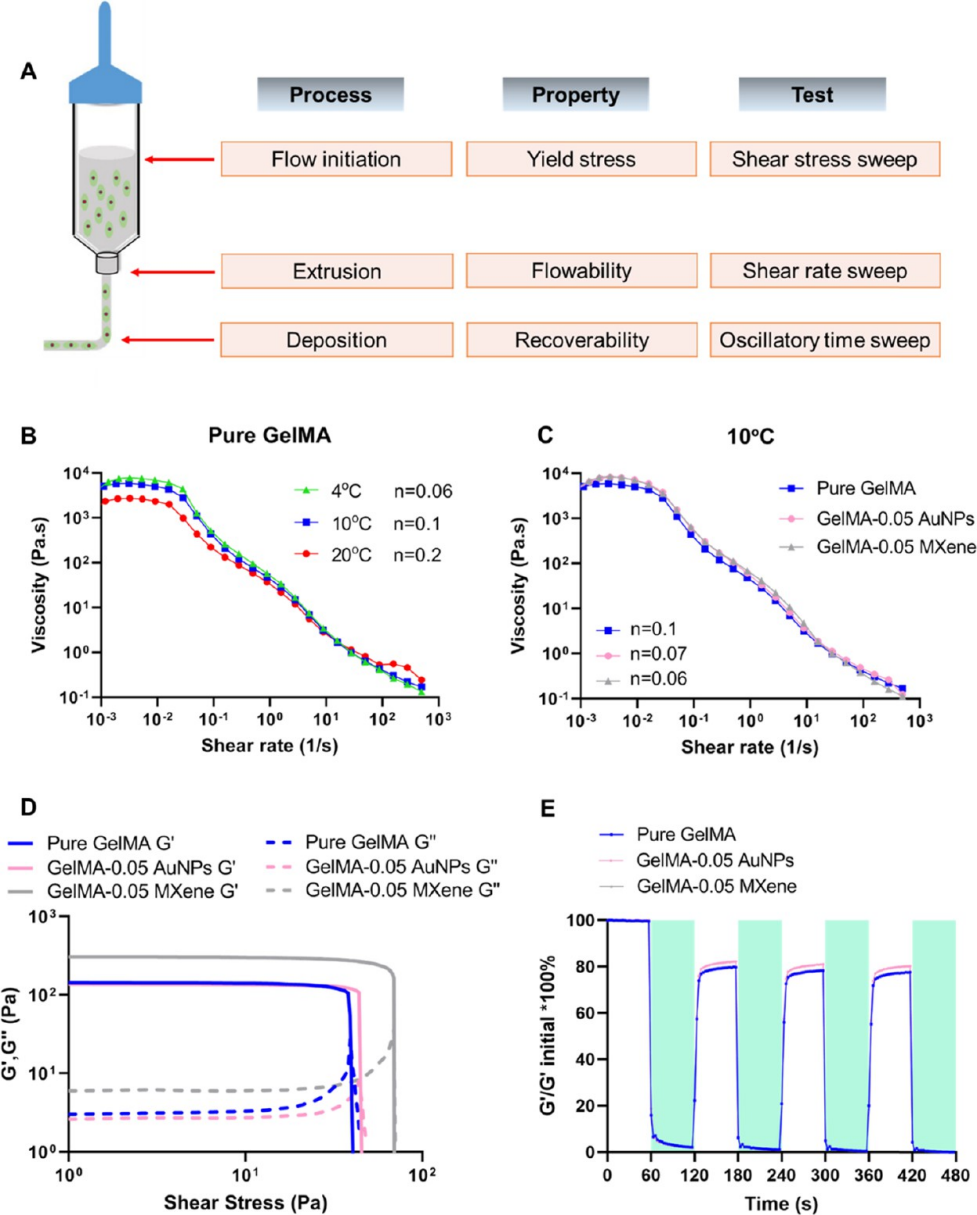
Rheological characterization of pure GelMA, GelMA-AuNPs, and GelMA-MXene hydrogels. (A) Schematic representation of the three main stages of extrusion bioprinting along with bioink property evaluated at each stage by the specified rheological test. (B) Log–log plot of viscosity vs shear rate obtained from the shear rate sweep test for pure GelMA at 4, 10, and 20 °C. (C) Log–log plot of viscosity vs shear rate obtained from the shear rate sweep test for GelMA-AuNPs and GelMA-MXene hydrogels at 10 °C. (D) Log–log plot of storage modulus *G*′ and loss modulus *G*″ vs shear stress obtained from the shear stress sweep test. (E) Oscillatory time sweep test for pure GelMA, GelMA- AuNPs, and GelMA-MXene hydrogels; shaded areas indicate high strain.

A variety of methods have been described in the scientific literature for determining the printability of bioinks, ranging from mere observations to quantification.^49−53^ The initial assessment of bioink printability includes fiber formation and layer stacking. However, a more comprehensive set of information can be obtained via rheological measurements and characterization. For the purpose of this work, shear-thinning and viscoelastic behaviors of bioinks were evaluated together with their recoverability after low–high strain-level cycles, as shown in Figure 5A.

Pure GelMA was subjected to shear rate sweep at 4, 10, and 20 °C. Results showed the typical non-Newtonian fluid behavior at all temperatures. For low shear rates, viscosity values are constant (zero-shear plateau) and drop significantly by increasing the shear rate. The viscosity–shear rate curves obtained were fitted with a simple power-law equation to derive the flow index of each material (*n*, exponent of the power law). This index is commonly used for differentiating and recognizing flow behaviors. In fact, a flow index of 1 is indicative of a Newtonian behavior, while values approaching 0 indicate non-Newtonian fluid with a higher degree of shear-thinning response.^54,55^ As expected, due to thermal gelation, the pure GelMA at 4 °C showed the highest viscosity at low shear rates and the lowest flow index (*n* = 0.06) compared to those obtained by the same bioink at 10 and 20 °C (*n* = 0.1 and *n* = 0.2, respectively), as shown in Figure 5B. Although the results at 4 °C are favorable, keeping cells at this temperature can negatively impact their viability. Hence, 10 °C was chosen as the optimal temperature for bioprinting, and the remaining part of the rheological characterization was performed at 10 °C only.

The addition of MXene and AuNPs to GelMA increased the viscosity of the bioink at a low shear rate and enhanced its shear-thinning behavior, as shown in Figure 5C. The resulting flow indices for GelMA-0.05 AuNPs and GelMA-0.05 MXene were 0.07 was 0.06, respectively. These values are comparable to the one obtained by pure GelMA at 4 °C. Since extrudability from the nozzle/needle is governed by the shear-thinning behavior of the bioink and it largely affects cell viability as well as its total printing time, these results indicated that the addition of MXene and AuNPs enhanced the extrudability of GelMA (e.g., decreased applied pressure needed to extrude the bioink from the nozzle/needle, decreased shear stress along the nozzle/needle, potential decrease in cell damage/death).^52^

Bioinks are viscoelastic materials and their mechanical response to applied stress can be divided into two components: elastic and viscous.^51^ In terms of moduli, this implies that two values are obtained from shear stress sweep tests, namely, storage modulus *G*′ and loss modulus *G*″. The bioinks under evaluation in this study showed pronounced elastic-like behavior at low shear stress levels. In fact, the initial storage moduli recorded during the tests were 1–2 orders of magnitude higher than loss moduli. As any other viscoelastic material, the ratio between the two modulus components tends to be 1 by increasing the shear stress test level. This point is known as yield stress and represents a key feature of bioinks since they must be thick enough to support any suspended cell and at the same time be able to flow as a liquid to be extruded in controlled conditions. Figure 5D shows that the addition of AuNPs and MXene increased the yield stress level of the pure GelMA alone. Among the three bioinks, the one with added MXene proved to have the highest yield stress, indicating improved filament formation and retention.

Bioinks face high strain levels while being extruded from the nozzle and low strain levels during deposition in the printing stage. It is important that the physical properties of a bioink are retained or recovered after undergoing a sudden change in the strain level. For this purpose, bioinks in this study were subjected to tests in an oscillatory mode alternating low (1%) and high (100%) strain levels every minute and this was repeated four times. A significant drop in the storage modulus was recorded for all bioinks while passing from a low to high strain level, as shown in Figure 5E. However, after the strain returned to 1%, *G*′ values were almost fully recovered every time. This finding indicates that GelMA with the tested formulations can revert to the initial condition of high viscoelastic gel once deposited by forming a stable structure.

**Figure 6:**
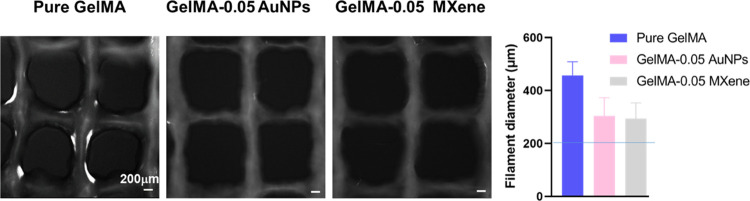
Printability of pure GelMA, GelMA-AuNPs, and GelMA-MXene hydrogels. Printed mesh constructs of pure GelMA, GelMA-AuNPs, and GelMA-MXene hydrogels along with filament diameter analysis.

To demonstrate the printability and to evaluate the printing fidelity of the tested bioink formulations, multilayered mesh constructs were printed and the filament diameter was assessed. Bioinks formed continuous filaments; however, the filament diameter was substantially enhanced upon the addition of AuNPs or MXene, as shown in Figure 6. In specific, the diameter of the filament printed using the 200 μm nozzle using GelMA-AuNPs and GelMA-MXene was smaller, closer to the diameter of the nozzle, compared to pure GelMA, demonstrating better printability. The printability of the formulated bioinks could be further enhanced via optimizing the printing speed and applied pressure.^14^ AuNP or MXene addition decreased filament spreading and irregularity, which led to an enhanced rectangular pore geometry (larger pore area with sharp edges).

### 3.5 Cell Viability and Differentiation of C2C12 Cells Encapsulated in Biocomposite Inks

During the bioprinting process, cells encapsulated in the bioink are subjected to various mechanical forces, including shear stress. Shear stress is considered the main cause of cell damage/death during the bioprinting process.^3,56^ To reduce its effect, bioinks with shear-thinning properties have been developed and have been shown to enhance maintenance of cellular viability.

**Figure 7:**
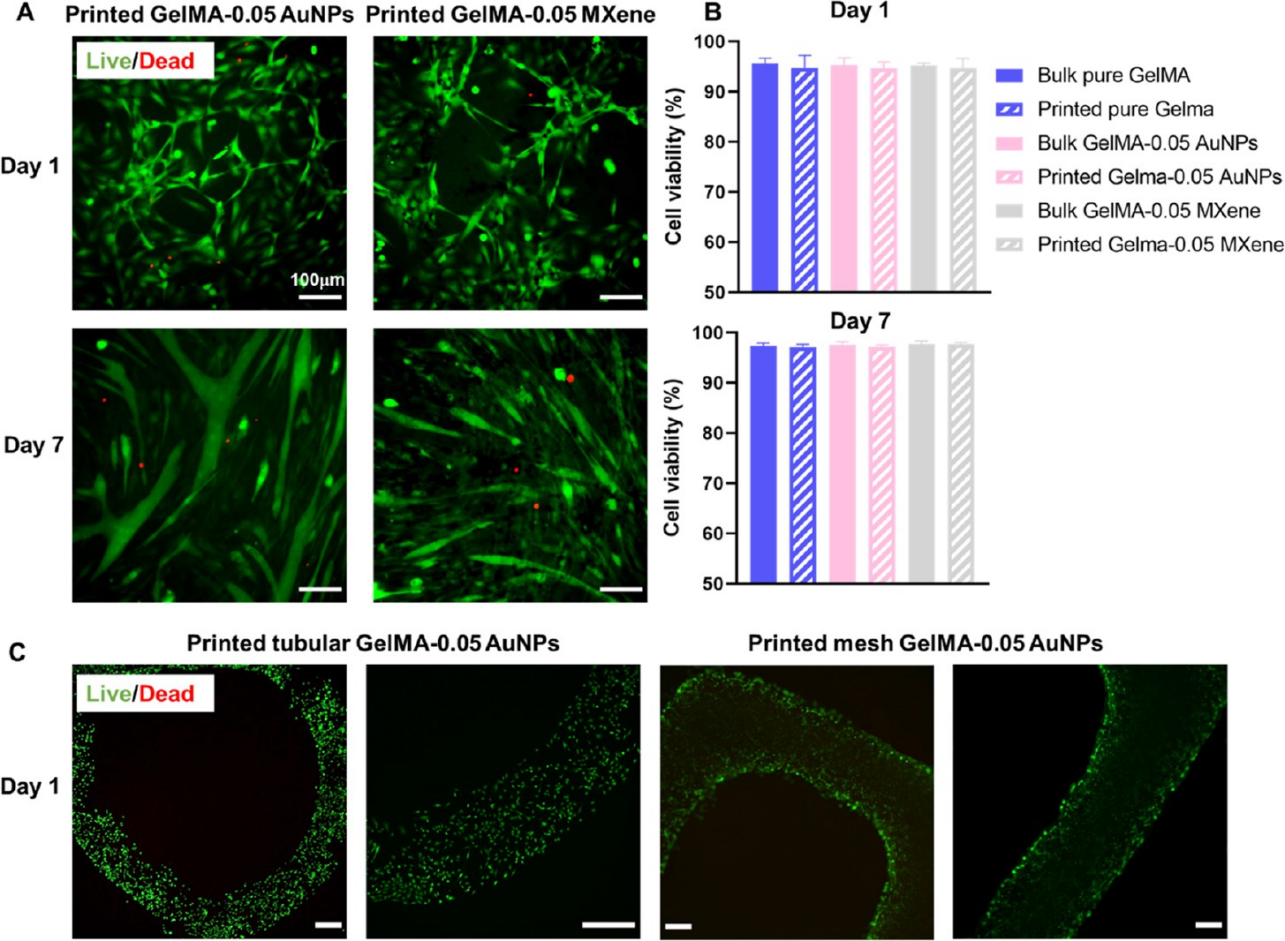
Bioprinted C2C12 using GelMA-AuNPs and GelMA-MXene bioinks. (A) Fluorescent microscopy images of cells bioprinted using GelMA-AuNPs and GelMA-MXene hydrogels. Red cells are dead cells, while green cells are live ones. (B) Viability of cells encapsulated in GelMA, GelMA-AuNPs, and GelMA-MXene hydrogels printed and nonprinted (bulk) on days 1 and 7. (C) Cells encapsulated in GelMA-AuNPs hydrogels bioprinted to produce different structures.

Rheological analysis of the GelMA-MXene or GelMA-AuNP hydrogels developed in this study demonstrates excellent shear-thinning properties, as described in Section 3.4. To assess whether such bioinks can maintain high cell viability, bioinks containing C2C12 cells were prepared, then either poured in 96-well plates (control) or printed at 10 °C, and finally assessed for the viability of their encapsulated cells on days 1 and 7. Bioprinted constructs with live cells stained green and dead cells stained red are shown in Figure 7. Viability analysis showed no significant decrease in cell viability between bulk and printed bioinks. Cells were homogeneously distributed in all hydrogels, as shown by the low-magnification images taken for segments of the tubular and mesh-like printed constructs.

**Figure 8:**
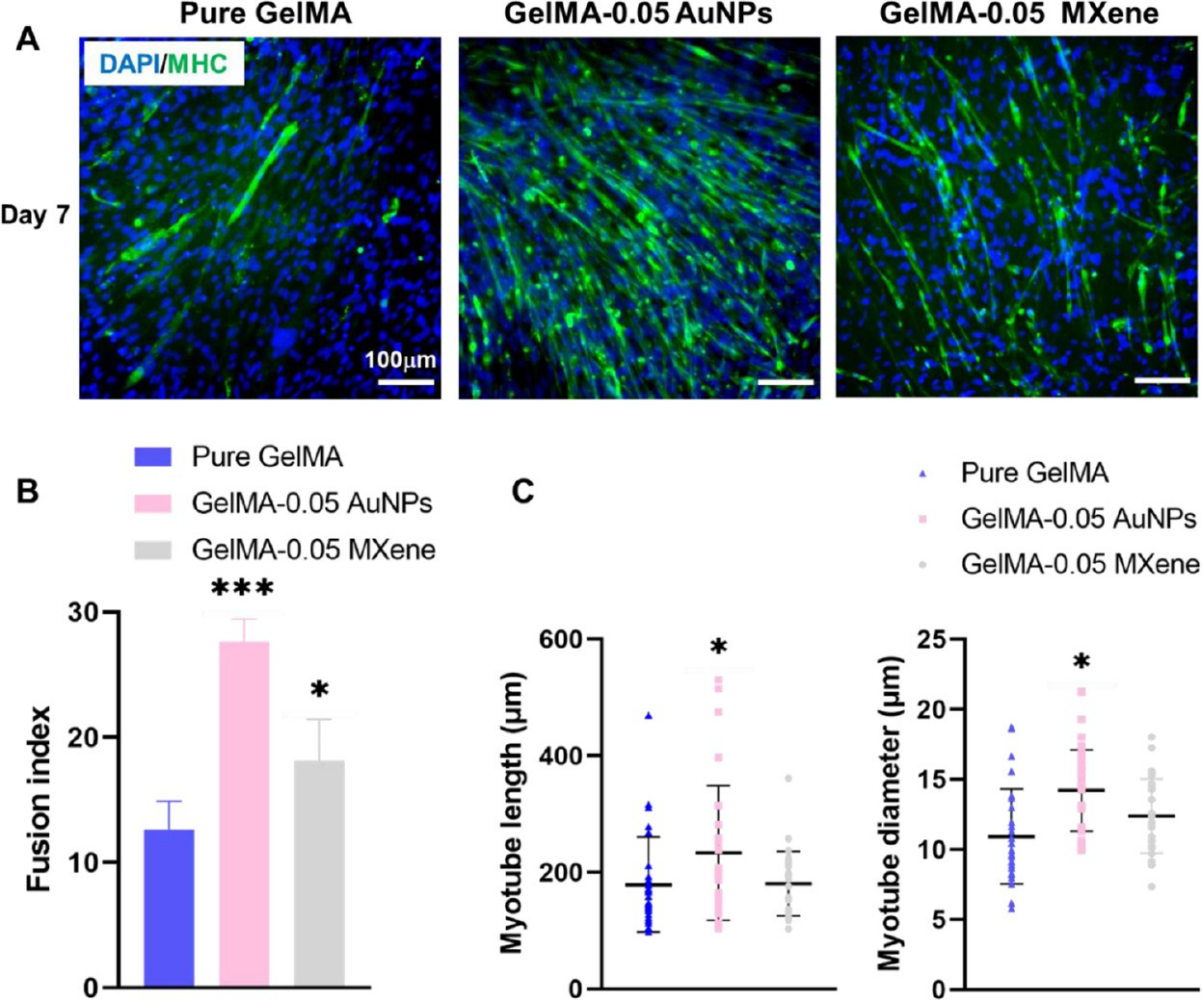
Differentiation of C2C12 cells encapsulated in pure GelMA, GelMA-AuNPs, and GelMA-MXene hydrogels. (A) Fluorescent microscopy images of cells encapsulated in GelMA-AuNPs and GelMA-MXene hydrogels and stained for the myosin heavy chain (MHC), (B) fusion index, and (C) myotube length and diameter for C2C12 cells encapsulated in pure GelMA, GelMA-AuNPs, and GelMA-MXene hydrogels.

Furthermore, to examine whether the addition of MXene and AuNPs has any effect on C2C12 differentiation, cells encapsulated in pure GelMA, GelMA-0.05 AuNPs, and GelMA-0.05 MXene were incubated in differentiation media for 1 week. On day 7, cells were stained for myosin heavy chain MHC, as shown in Figure 8A. Consequently, the fusion index, the length of myotubes, and the diameter of myotubes were analyzed. The fusion index represents the number of nuclei inside MHC-positive myotubes (if 2 or more) to the number of total nuclei, and it is an indicator of the ability of single nucleated myoblast to fuse and form multinucleated myotubes.^57,58^ As shown in Figure 8B, the fusion index increased from 12.65 ± 2.23% for pure GelMA to 27.64 ± 1.80% for GelMA-0.05 AuNPs and to 18.12 ± 3.31% for GelMA-0.05 MXene. The average length of myotubes and the average diameter were significantly increased when cells were encapsulated in GelMA-0.05 AuNPs compared to pure GelMA and GelMA-0.05 MXene, as shown in Figure 8C. These results indicate that MXene and AuNP additives enhance skeletal muscle differentiation in GelMA hydrogels.

We attribute the enhancement in C2C12 differentiation to the fact that the GelMA-MXene and GelMA-AuNPs are electrically conductive bioinks. Electrical conductivity is known to promote electrical communication between skeletal muscle cells, induce myogenic differentiation, and accelerate maturation.^59−62^ Further enhancement is expected upon electrical stimulation of cells as both bioinks conduct electrical signals, which make them promising candidates in skeletal muscle tissue engineering.

## 4 Conclusions

In this study, we optimized low-concentration GelMA hydrogels for 3D bioprinting of skeletal muscle tissue. We demonstrated that 2% GelMA cross-linked for 4 min produced optimal cellular elongation and spreading. However, such a hydrogel suffered from poor printability at room temperature and lacked conductivity, a property essential for skeletal muscle cells. Therefore, here, we enhanced the printability and conductivity of 2% GelMA cross-linked for 4 min by incorporating it with either gold nanoparticles or MXene nanosheets. Our results demonstrated that incorporating MXene and AuNPs into low-concentration GelMA followed by thermal cross-linking at 10 °C significantly improved the rheological properties of the hydrogel and endowed GelMA with conductivity. In particular, the excellent shear-thinning properties that resulted from the addition of MXene or AuNPs to GelMA enhanced bioink extrudability, printability, and shape recovery and shielded cells from process-induced stresses. In addition, Au nanoparticles and MXene nanosheets by their inherent conductive properties enhanced the capability of GelMA in conducting electrical signals and promoted C2C12 differentiation even without any electrical stimulation. Interestingly, addition of AuNPs and MXene nanosheets to GelMA, at the chosen concentrations, did not significantly affect the mechanical stiffness of GelMA, and therefore, cells maintained the regular morphology seen in pure GelMA in 3D. Taken together, these results demonstrate the potential of GelMA-AuNPs and GelMA-MXene bioinks in tissue engineering applications, specifically as biocompatible and biomimetic bioinks with enhanced printability and conductivity.

Future work will include a deeper investigation of the effect of different concentrations of MXene and AuNPs on the cross-linking kinetics of GelMA along with its rheology. In addition, it will include the utilization of the developed conductive bioinks in exogenous electrical stimulation studies that aim to examine cell contraction, development, orientation, and maturation.
